# Serological Cross-Reactivity between Merkel Cell Polyomavirus and Two Closely Related Chimpanzee Polyomaviruses

**DOI:** 10.1371/journal.pone.0097030

**Published:** 2014-05-09

**Authors:** Jérôme T. J. Nicol, Etienne Liais, Romain Potier, Elisa Mazzoni, Mauro Tognon, Pierre Coursaget, Antoine Touzé

**Affiliations:** 1 Université François Rabelais, Virologie Immunologie Moléculaires, Tours, France; 2 INRA UMR 1282, Infectiologie et Santé Publique, Tours, France; 3 Association Beauval Nature pour la Conservation et la Recherche, Saint Aignan sur Cher, France; 4 Department of Morphology, Surgery and Experimental Medicine, School of Medicine, University of Ferrara, Ferrara, Italy; Penn State University School of Medicine, United States of America

## Abstract

Phylogenetic analyses based on the major capsid protein sequence indicate that Merkel cell polyomavirus (MCPyV) and chimpanzee polyomaviruses (PtvPyV1, PtvPyV2), and similarly Trichodysplasia spinulosa-associated polyomavirus (TSPyV) and the orangutan polyomavirus (OraPyV1) are closely related. The existence of cross-reactivity between these polyomaviruses was therefore investigated. The findings indicated serological identity between the two chimpanzee polyomaviruses investigated and a high level of cross-reactivity with Merkel cell polyomavirus. In contrast, cross-reactivity was not observed between TSPyV and OraPyV1. Furthermore, specific antibodies to chimpanzee polyomaviruses were detected in chimpanzee sera by pre-incubation of sera with the different antigens, but not in human sera.

## Introduction

Twelve polyomaviruses have been indentified in humans, including the well-known BKPyV and JCPyV discovered in the early 1970s, KIPyV and WUPyV, isolated in 2007 from pulmonary secretions [1], [2]. Merkel cell polyomavirus (MCPyV) was identified in 2008 in Merkel cell carcinoma [3]. Trichodysplasia spinulosa-associated polyomavirus (TSPyV) was identified in skin lesions of a transplant patient suffering from Trichodysplasia spinulosa [4]. Three human polyomaviruses named human polyomaviruses 6, 7 and 9 (HPyV6, HPyV7 and HPyV9) were discovered on normal skin [5]–[7]. In addition, three polyomaviruses were recently identified in the gastro intestinal tract, i.e. Malawi Polyomavirus (MWPyV) and Saint-Louis polyomavirus (STLPyV) found in children's stools [8], [9], and human polyomavirus 12 (HPyV12) found in liver samples [10]. HPyV6, 7, 9, 12, MWPyV, and STLPyV have not so far been associated with any human diseases.

Serological studies have shown that most adults have had early exposure to human polyomaviruses [11]–[17]. In addition, several studies have indicated the absence of cross-reactivity between the different human polyomaviruses, with the exception of low cross-reactivity observed between HPyV6 and HPyV7 [15], [17]. Antigenic similarities between human and simian polyomaviruses have been described, particularly between simian virus (SV40) and BKPyV and JCPyV [18]–[20]. Competitive inhibition in ELISA [18], [19], [21] and neutralization assays [22] have shown that reactivity to SV40 in humans is due to cross-reaction between SV40, BKPyV and JCPyV. Moreover, cross-reactivity between the newly discovered HPyV9 and the simian lymphotropic polyomavirus (LPyV) has recently been reported [23], [24], explaining why many years ago around 15–30% of humans were LPyV-seropositive [19], [25].

**Figure 1:**
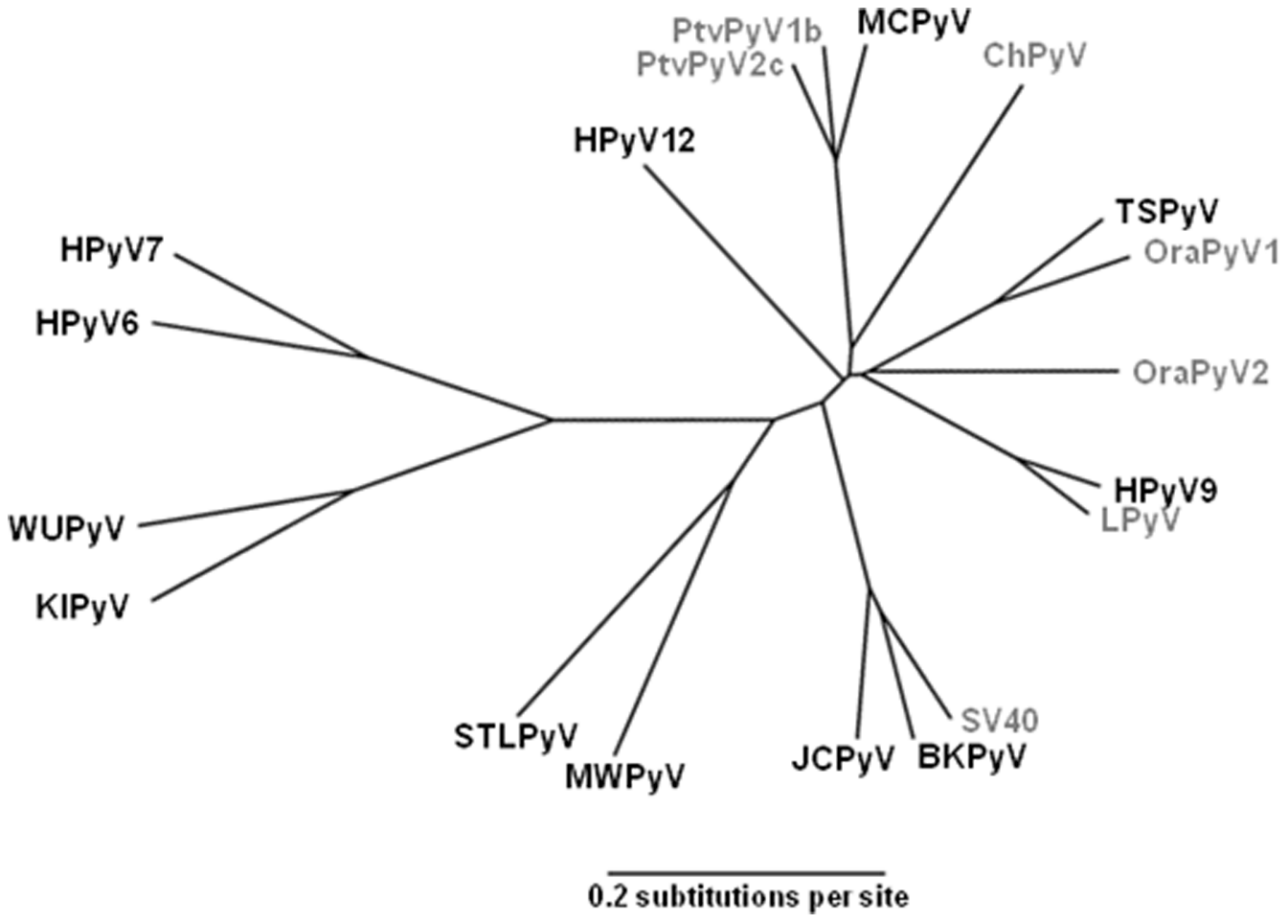
Phylogenetic relationships between human and closely related simian polyomaviruses based on VP1 protein. Human polyomaviruses are in black and simian polyomaviruses are in gray. Sequence accession numbers used are NC_001538 for BKPyV, NC_001699 for JCPyV, NC_009238 for KIPyV, NC_009539 for WuPyV, NC_010277 for MCPyV, NC_014406 for HPyV6, NC_014407 for HPyV7, NC_014361 for TSPyV, HQ696595 for HPyV9, NC_018102 for MWPyV, JX463183 for STLPyV, JX308829 for HPyV12, NC_001669 for SV40, NC_004763 for LPyV, AY691168 for ChPyV, HQ385747 for PtvPyV1b, HQ385750 for PtvPyV2c, FN356900 for OraPyV1 and FN356901 for OraPyV2.

As MCPyV is phylogenitically close to recently discovered chimpanzee polyomaviruses (PtvPyV1 and PtvPyV2 [26]) and similarly TSPyV is closely related to orangutan polyomavirus 1 (OraPyV1 [27]) (Figure 1), we investigated in this study the possible existence of cross-reactivity between these closely related polyomaviruses.

## Materials and Methods

### Serum samples

The seroprevalence of chimpanzee polyomaviruses was investigated in 828 1- to 100-year-old subjects from the city of Ferrara, Italy. This population had previously been investigated for MCPyV and TSPyV antibodies [15] and the serum samples were obtained from the Blood Center and from the Clinical Analysis Laboratory, University Hospital of Ferrara, Italy, using a protocol approved by the County Ethical Committe, University Hospital of Ferrara, Ferrara, Italy. Consent from participants was not requested for polyomavirus testing since samples were de-identified and analyzed anonymously with indications of age and gender, only. Samples were stored at −20°C until tested. The seroprevalence of OraPyV1 was investigated in a subset of 300 of these samples.

In addition, serum samples from thirteen chimpanzees, two orangutans and one gorilla were investigated for MCPyV, PtvPyV1, PtvPyV2, TSPyV and OraPyV1 antibodies. Samples from these great apes were collected during routine health checks by zoo veterinarians (ZooParc de Beauval, Saint-Aignan, France). No animal was specifically sampled for the present study and samples were a donation from the ZooParc. Housing conditions, feeding regiments and environmental enrichment fulfilled all the requirements of the European Association of Zoos and Aquaria (EAZA). Great apes in this study are housed in multi-male/multi-female age stratified groups to resemble the wild population and are fed a balanced diet that includes a mixture of vegetables, fruits, and nutritionally complete dry food. The presentation of food is an obvious form of enrichment, and can help increase foraging time and decrease aggression and abnormal behaviour. Moreover, structures are designed to allow the animals to climb to obtain food. Different objects are available to the animals both in exhibit and off-exhibit areas. Enclosures contain morphology specific furniture, climbing structures, trees, large rocks, shade and weather shelters, hiding places, or to engage in climbing, swinging on limbs and vines, arboreal play, to ensure the normal physical development. A fulltime team of veterinarians is present at the Beauval zoo and physical examination of all animals in a group is realised at 18–24 month intervals in the dedicated veterinary hospital within the zoo.

### Production of Virus-Like Particles

**Figure 2:**
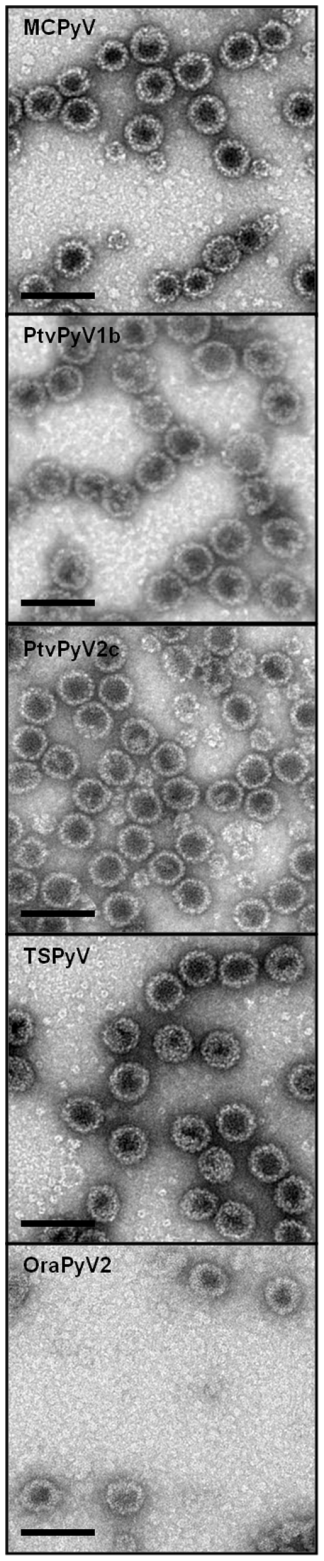
Electron micrographs of VP1 virus-like particles for MCPyV, PtvPyV1b, PtvPyV2c, TSPyV and OraPyV1. VP1 were produced using recombinant baculoviruses. The preparations were applied to carbon-coated grids, negatively stained with 1.5% uranyl acetate and observed at 50,000 nominal magnification with a JEOL 1011 electron microscope (Scale bars, 100 nm).

Production of MCPyV and TSPyV VLPs in insect cells has been described previously [15], [28]. VLPs were also generated for PtvPyV1, PtvPyV2 and OraPyV1. Briefly, VP1 coding sequences were obtained by total synthesis with codon usage-adapted sequences for expression in *Spodoptera frugiperda* cells (Genscript, Piscataway, NJ, USA) (Sequences based on PtvPyV1b (HQ385747), PtvPyV2c (HQ385750) and OraPyV1 (FN356900)) [26], [27]. The different VP1 genes were cloned under the control of the polyhedrin promoter of pFastBac Dual plasmid (Invitrogen, FisherScientific, Illkirch, France) and then used to generate recombinant baculoviruses using the Bac-to-Bac system (Invitrogen). HiFive cells maintained in Grace medium (Invitrogen) were infected with the different recombinant baculoviruses for production of the polyomavirus VLPs. VLPs were then purified by ultracentrifugation (18 h at 30,000 rpm in a Beckman SW 32 rotor) in a CsCl gradient and assembly of VP1 into VLPs was verified by electron microscopy. The preparations were applied to carbon grids, negatively stained with 1.5% uranyl acetate and observed with a JEOL 1011 electron microscope at 50,000 nominal magnification (Figure 2).

### Detection of polyomavirus antibodies

To detect antibodies against polyomaviruses, virus-like particle-based enzyme immunoassays similar to the assays developed for MCPyV, LPyV and HPyV9 were used [24], [28]. Briefly, ELISAs were performed in microplates coated with 100 ng of extract enriched for VLPs. VLP concentrations were determined using the Qubit Protein Assay Kit (Invitrogen). Sera were tested at 1∶100 dilution and peroxidase-conjugated goat anti-human IgG (Southern Biotech, Clinisciences, Nanterre, France) diluted 1: 20,000 was used to detect binding of human and great ape IgGs. The cut-off values were set at 0.200, determined as previously [24], [28].

### Characterization of cross-reactivity between polyomaviruses

To assess the degree of antigenic cross-reactivity between MCPyV and PtvPyV1 and PtvPyV2 VLPs, competition assays were performed as described previously [24] by pre-incubation (1 h at 37°C) of selected positive sera (diluted 1∶100) with buffer containing twenty times the coating amount of MCPyV, PtvPyV1 or PtvPyV2 VLPs. Cut-off for competition was set for more than 60% inhibition after pre-incubation. Human and chimpanzee sera used for competition assays were selected from individuals with high optical density (OD) values to the three polyomaviruses investigated.

### Bioinformatic analysis

The phylogenetic tree is based on major capsid VP1 protein sequences aligned using ClustalW (www.genome.jp/tools/clustalw). The tree was obtained by a neighbor-joining method using ClustalW2phylogeny (www.ebi.ac.uk/Tools/phylogeny), and graphic display was performed using Figtree.

Protein identity between the different VP1 proteins was determined using T-Coffee (http://www.ebi.ac.uk/Tools/msa/tcoffee/).

### Statistical methods

Cross-reactivity between human and great ape polyomaviruses was evaluated by correlation analysis between OD values for the different polyomaviruses investigated using Spearman coefficient correlation (XLStat software, Addinsoft, Paris, France).

## Results

### Antibodies to MCPyV, PtvPyV1, PtvPyV2, TSPyV and OraPyV1 in humans and great apes

**Figure 3:**
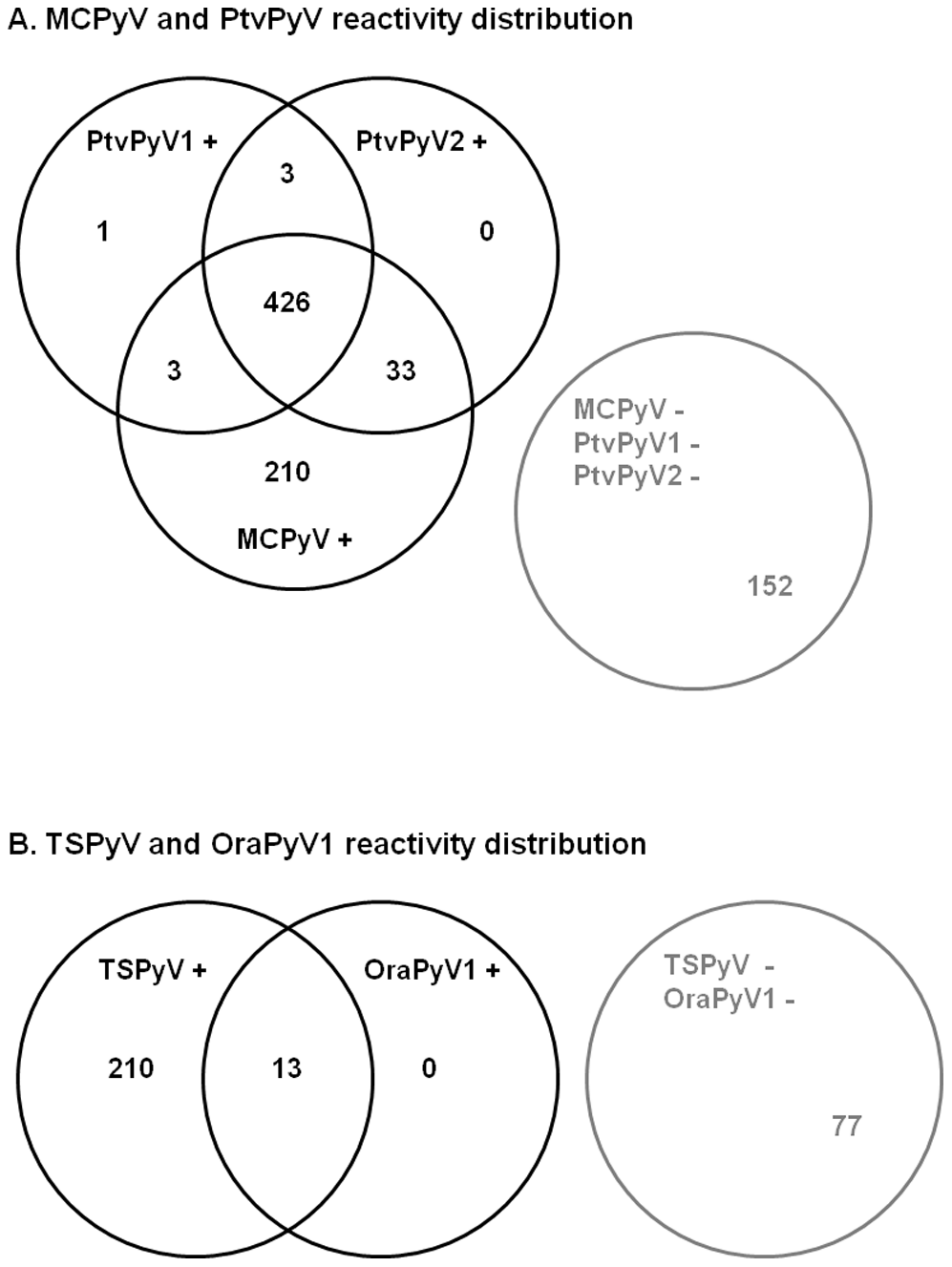
Human seroreactivity distribution Venn diagrams. (A) Negative (−), simple, and multiple seropositive (+) sera for 828 sera investigated for MCPyV, PtvPyV1 and PtvPyV2. (B) Negative (−), simple and double seropositive (+) sample for the 300 sera investigated for TSPyV and OraPyV.

**Figure 4:**
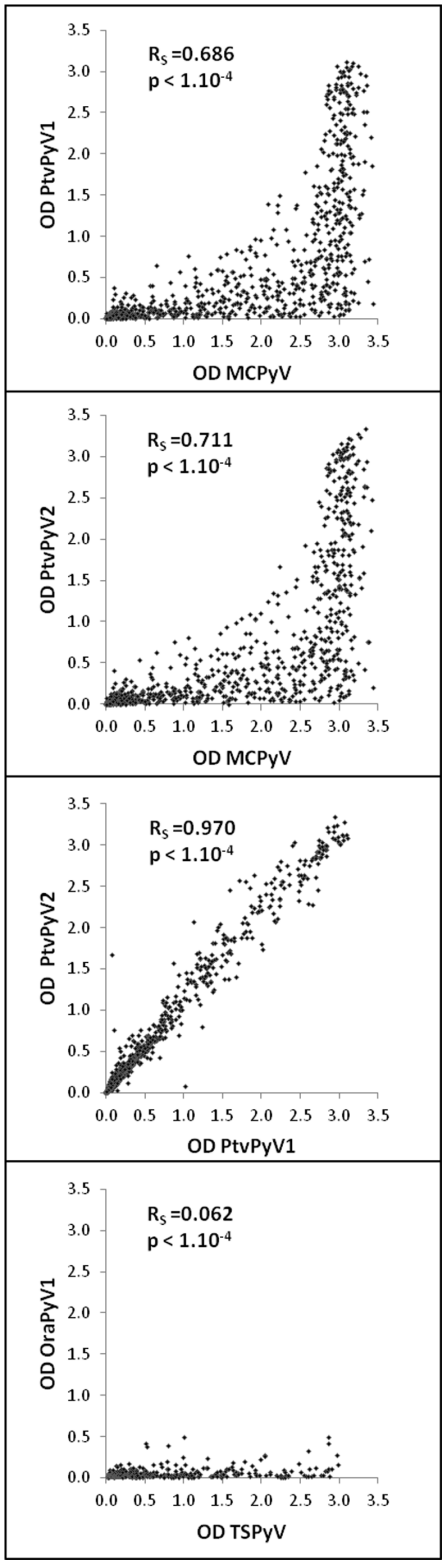
Correlation between MCPyV and PtvPyV reactivity and between TSPyV and OraPyV1 reactivity. Correlation between ELISA reactivity of human serum samples against the different VLPs. Each point represents one serum sample. Correlation coefficients (Spearman) were determined using XLStat software.

Of the 828 subjects investigated, 433 (52.3%) were found to be seropositive for PtvPyV1 and 462 (55.8%) for PtvPyV2. It should be noted that 429 subjects were found to be positive for both PtvPyV1 and PtvPyV2, while the ELISA OD value was found to be close to the cut-off value for the remaining 37 discordant results. In addition, the positive sera for PtvPyV were also found to be positive for MCPyV (Figure 3). Correlation analysis between PtvPyV1, PtvPyV2 and MCPyV reactivity was performed with the non-parametric Spearman test (Figure 4). Serological identity between PtvPyV1 and PtvPyV2 was observed (R_S_ = 0.970, p<1.10^−4^) and a strong correlation was found between MCPyV and the two PtvPyVs (R_S_ = 0.686, p<1.10^−4^, and R_S_ = 0.711, p<1.10^−4^).

Antibodies against OraPyV1 were detected in 13 of the 300 human (4.3%) sera investigated, although antibodies against TSPyV were detected in 223 of them (74.3%) (Figure 3). It should be noted that reactivity of human sera with OraPyV1 VLPs was weak, the mean OD value for positive samples being 0.339 (0.216 to 0.494). Correlation analysis indicated no evidence of cross-reactivity between these two polyomaviruses (Rs = 0.062, p<1.10^−4^) (Figure 4).

MCPyV, PtvPyV1, PtvPyV2, TSPyV and OraPyV1 antibodies were also investigated in sera from 16 great apes (13 chimpanzees, 2 orangutans, 1 gorilla) (Table 1). MCPyV, PtvPyV1 and PtvPyV2 antibodies were detected in 6 chimpanzees (46.1%) and in the gorilla, but not in the two orangutans. OraPyV1 antibodies were detected in both orangutans and in only one chimpanzee (7.7%). TSPyV antibodies were detected in 8 chimpanzees (61.5%) and in the gorilla.

**Table 1:** Detection of antibodies to MCPyV, PtvPyV1, PtvPyV2, TSPyV and OraPyV1 polyomaviruses in 16 great apes.

Great ape	MCPyV	PtvPyV1	PtvPyV2	TSPyV	OraPyV1
Chimp1	+	+	+	+	−
Chimp2	−	−	−	−	−
Chimp3	−	−	−	+	−
Chimp4	−	−	−	−	−
Chimp5	+	+	+	+	−
Chimp6	+	+	+	+	+
Chimp7	−	−	−	−	−
Chimp8	−	−	−	+	−
Chimp9	+	+	+	+	−
Chimp10	−	−	−	+	−
Chimp11	−	−	−	−	−
Chimp12	+	+	+	−	−
Chimp13	+	+	+	+	−
Oran1	−	−	−	−	+
Oran2	−	−	−	−	+
Gorilla1	+	+	+	+	−

### Cross reactivity between MCPyV, PtvPyV1 and PtvPyV2 VLPs

To assess the degree of antigenic cross-reactivity between MCPyV, PtvPyV1 and PtvPyV2, competition assays were performed by pre-incubation of sera with twenty times the coating amount of MCPyV, PtvPyV1 or PtvPyV2 VLPs.

**Figure 5:**
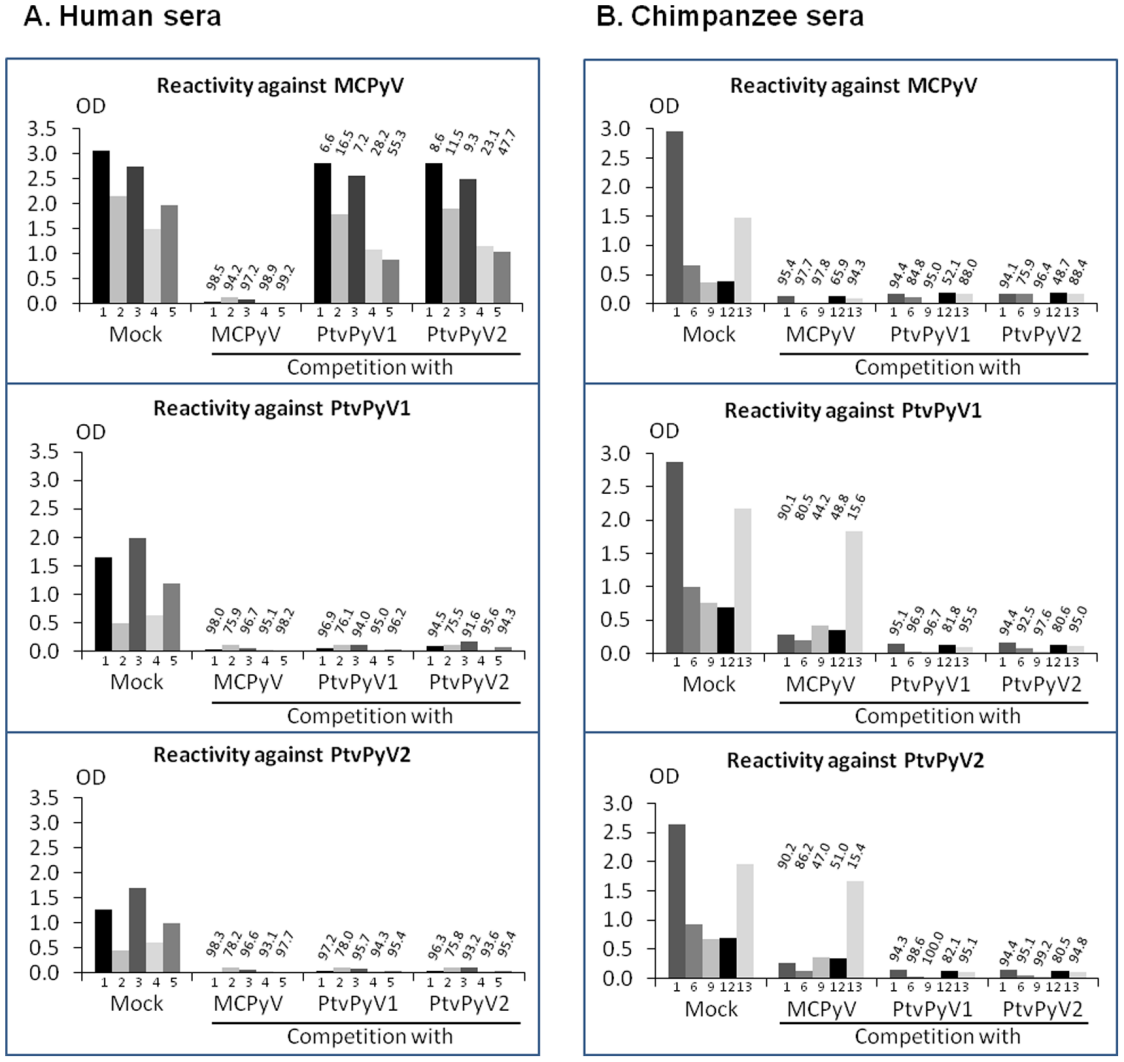
Competitive inhibition of seroreactivity between MCPyV, PtvPyV1 and PtvPyV2. ELISA Reactivity against MCPyV, PtvPyV1 and PtvPyV2 was determined for five human (A) and five chimpanzee (B) sera after preincubation with or without MCPyV, PtvPyV1 or PtvPyV VLPs. Percentage of inhibition after preincubation is indicated above bars.

In MCPyV ELISA, the reactivity of the human sera was inhibited by preincubation with MCPyV VLPs, but not with PtvPyV1 or PtvPyV2 VLPs (Figure 5A). The serum reactivity in PtvPyV1 and PtvPyV2 ELISAs was inhibited by preincubation with MCPyV, PtvPyV1 and PtvPyV2 VLPs. These results clearly indicate that the PtvPyV1 and PtvPyV2 reactivity detected in human sera was due to MCPyV infection and not to infection with the chimpanzee polyomaviruses.

The specificity of the antibodies detected in chimpanzees was also investigated by similar competition assays for five chimpanzee sera. In MCPyV ELISAs, the reactivity of the 5 chimpanzee sera investigated (Figure 5B) was inhibited by preincubation with PtvPyV1 and PtvPyV2 VLPs, as well as with MCPyV VLPs. In PtvPyV1 and PtvPyV2 ELISAs, 80.6 to 100% of reactivity was inhibited after preincubation with PtvPyV1 and PtvPyV2 VLPs, indicating that the five chimpanzees were infected with PtvPyV1 and/or PtvPyV2. For three chimpanzee sera (Chimp 9, 12 and 13) the reactivity was not inhibited by preincubation with MCPyV. The results clearly indicated the absence of MCPyV infection in these animals. In contrast, for the other two chimpanzee sera (Chimp 1 and 6), 80.5 to 90.1% of the reactivity was inhibited by pre-incubation with MCPyV VLPs, suggesting the possibility that these two animals had also been infected with MCPyV.

## Conclusions

Antibodies against PtvPyV1 and PtvPyV2 were detected in a high percentage of humans (52.3% and 55.8%, respectively), with similar OD values observed for both viruses. Correlation analysis confirmed these results (R_S_ = 0.970, p<1.10^−4^), suggesting that these two viruses belong to the same serotype. In addition, comparison of the PtvPyV1 and PtvPyV2 results with anti-MCPyV serology data suggested a high degree of cross-reactivity between these chimpanzee polyomaviruses and MCPyV (R_S_ = 0.686, p<1.10^−4^ and R_S_ = 0.711, p<1.10^−4^). These findings are in agreement with the phylogenetic analysis of polyomavirus VP1 sequences (Figure 1) and the high VP1 protein identity between these viruses (PtvPyV1-PtvPyV2, 85.6%; PtvPyV1-MCPyV, 84.8%; PtvPyV2-MCPyV, 84.1%). To confirm the cross-reactivity between these viruses, and to investigate whether chimpanzees and humans could be infected with polyomaviruses from other species, human and chimpanzee samples were investigated for reactivity against these three polyomaviruses after pre-incubation with an excess of VLPs. The results clearly indicated that the seropositivity to PtvPyV1 and PtvPyV2 observed in humans was due to cross-reactivity of these viruses with MCPyV, and suggested that no infection with these two chimpanzee polyomaviruses might occur in humans.

The findings indicated specific infection with PtvPyV1 and/or PtvPyV2 in 46.1% of the chimpanzees. PtvPyV1 and PtvPyV2 infections are very common, as reported for other chimpanzee polyomaviruses (ChPyV, PtvPyV3, PtvPyV4 and PtsPyV2) [29], [30]. In addition, pre-incubation studies suggested the possibility that MCPyV infection occurred in two chimpanzees. It should be noted that these chimpanzees are in close contact with humans (veterinary and animal handlers), and thus it is possible that such infections might not be observed in wild animals.

In contrast to the findings obtained with PtvPyV1 and PtvPyV2, only a few human sera (4.3%) reacted with OraPyV1 VLPs, and correlation analysis indicated an absence of cross-reactivity between TSPyV and OraPyV1 (R_S_ = 0.062, p<1.10^−4^), although phylogenetic analysis of VP1 sequences (Figure 1) and protein identity of 79.8% suggest that these viruses are very close. Similarly, no cross-reactivity was reported between human polyomaviruses and four other chimpanzee polyomaviruses (ChPyV, PtvPyV3, PtvPyV4 and PtsPyV2) whereas human sera were frequently found to be positive for these chimpanzee polyomaviruses [30]. The circulation of an as yet unknown human polyomavirus closely related to OraPyV1 could be an explanation for the low number of humans seropositive for OraPyV1.

In addition, anti-TSPyV antibodies were detected in 8 of the 13 chimpanzees investigated, suggesting that these great apes in close contact with humans had been infected by TSPyV. It is also possible that these animals had been infected with an as yet unknown chimpanzee polyomavirus closely related to TSPyV. It must also be noted that only one chimpanzee was anti-OraPyV1 positive, suggesting that this orangutan polyomavirus does not circulate widely in chimpanzees.

In conclusion, our findings demonstrated the existence of cross reactivity between MCPyV and PtvPyVs, but no evidence of human infection with PtvPyV polyomaviruses. However, no evidence of cross-reactivity was found between TSPyV and OraPyV1, suggesting that anti-TSPyV antibodies in humans are specific of TSPyV infection. Cross-reactivity between human and simian polyomaviruses is probably the result of host and virus co-evolution from a common ancestor.
